# B-Cell Acute Lymphoblastic Leukaemia Presenting With Bilateral Renal Enlargement

**DOI:** 10.7759/cureus.90721

**Published:** 2025-08-22

**Authors:** Omolayo Soruna, Muniba Qureshi, Mani Prabha, Jack Bartram, Eva Tsouana

**Affiliations:** 1 Paediatrics and Child Health, Mid and South Essex NHS Foundation Trust, Essex, GBR; 2 Paediatric Radiology, Mid and South Essex NHS Foundation Trust, Essex, GBR; 3 Paediatric Oncology, Great Ormond Street Hospital, London, GBR; 4 Paediatrics, Mid and South Essex NHS Foundation Trust, Essex, GBR

**Keywords:** acute lymphoblastic leukaemia, b cell all, bilateral nephromegaly, paediatric malignancy, renal enlargement

## Abstract

Acute lymphoblastic leukaemia (ALL) is the most common paediatric malignancy. While extramedullary manifestations can occur, renal infiltration is rare. We report the case of a previously healthy one-year-old girl who presented with a two-week history of intermittent fever, lethargy, and progressive abdominal distension. On examination, she was lethargic, with bilateral palpable abdominal masses. Laboratory investigations revealed leukocytosis, bicytopenia, and abnormal urinalysis. A peripheral blood film and flow cytometry confirmed B-cell ALL. Ultrasonography demonstrated bilateral renal enlargement, suggestive of leukemic infiltration. Despite massive nephromegaly, she maintained normal renal function, fluid balance, blood pressure, and urine output. Her nephromegaly resolved clinically within four weeks of initiation of multi-agent chemotherapy. Renal involvement in ALL is an uncommon extramedullary manifestation, often asymptomatic and incidentally discovered on imaging. The majority of reported cases show that leukemic infiltration of the kidneys typically does not impair renal function. Nephromegaly in ALL has no established prognostic significance, with most cases resolving following chemotherapy initiation. This case highlights the importance of considering ALL in the differential diagnosis of paediatric patients presenting with unexplained nephromegaly and/or abdominal masses. Although renal dysfunction in this context is rare, close monitoring and careful management to prevent tumor lysis syndrome are of paramount importance to optimise outcomes.

## Introduction

Acute lymphoblastic leukaemia (ALL) is the most common type of cancer in paediatric patients, accounting for about 25% of cancer diagnoses in children under 15 years old, with the highest incidence in those aged 0-4 years [1-2]. It is characterized by the proliferation of immature lymphoid cells in the bone marrow, peripheral blood, and other organs. The majority of childhood ALL cases are of B-cell lineage. 

ALL often presents with non-specific symptoms, which may include fatigue, fever, recurrent infections, anaemia, bone pain, hepatomegaly, and splenomegaly [3]. Renal enlargement at the time of diagnosis is a rare presentation, with only a few reported cases [4]. 

We report the case of a young girl who presented with intermittent fevers and was found to have bilateral enlarged abdominal masses on examination. 

## Case presentation

A previously healthy one-year-old girl presented with a two-week history of intermittent fever, cough, and cold. She had been treated by her general practitioner with paracetamol and a seven-day course of oral amoxicillin, but there was no improvement in her fever. Over the preceding days, she became increasingly lethargic and intermittently irritable. Her mother also noticed new-onset abdominal distension, though this was not associated with vomiting, loose stools, or urinary symptoms. 

Her initial observation showed a temperature of 36.9 ^o^C, respiratory rate of 40 breaths per minute, heart rate of 146 beats per minute, and blood pressure of 110/57 mmHg, with a mean blood pressure of 73 mmHg. On physical examination, she appeared pale and lethargic but was rousable. Her chest was clear, and heart sounds were normal. There was no palpable lymphadenopathy. Abdominal examination revealed significant distension with two palpable masses, though it was not possible to palpate above the masses. 

Laboratory investigations revealed leukocytosis, bicytopenia, and elevated lactate dehydrogenase (LDH), as shown in Table 1. The clotting profile was within normal limits. Her initial blood film showed significant anaemia, thrombocytopenia, lymphocytosis with circulating blast cells. Urine microscopy revealed a raised white blood cell (1954 x 10^6^/L (>100 WBC x 10^6^ is significant and suggestive of infection) and red blood cell (RBC) 221 x 10^6^/L (<10 RBC x 10^6^/L equates to trace RBC), with all other parameters within normal range and no growth on culture.

**Table 1:** Laboratory test results

Blood Test (Units)	Patient Value	Reference Range
Haemoglobin (g/L)	69	100 – 135
White cell count (x 10^9^/L)	44.7	6 – 18
Platelets (x 10^9^/L)	25	150 – 400
Neutrophils (x 10^9^/L)	2.91	1 – 8.5
Lymphocytes (x 10^9^/L)	41.12	3 – 13.5
Sodium (mmol/L)	142	133 – 146
Potassium (mmol/L)	4.1	3.5 – 5.3
Urea (mmol/L)	3.1	2.5 – 6.5
Creatinine (umol/L)	35	15 – 36
Magnesium (mmol/L)	0.87	0.7 – 1.0
Phosphate (mmol/L)	1.5	1.3 – 2.4
Uric Acid (umol/L)	496	140 – 360
Lactate Dehydrogenase (U/L)	> 15000	240 – 480

**Figure 1:**
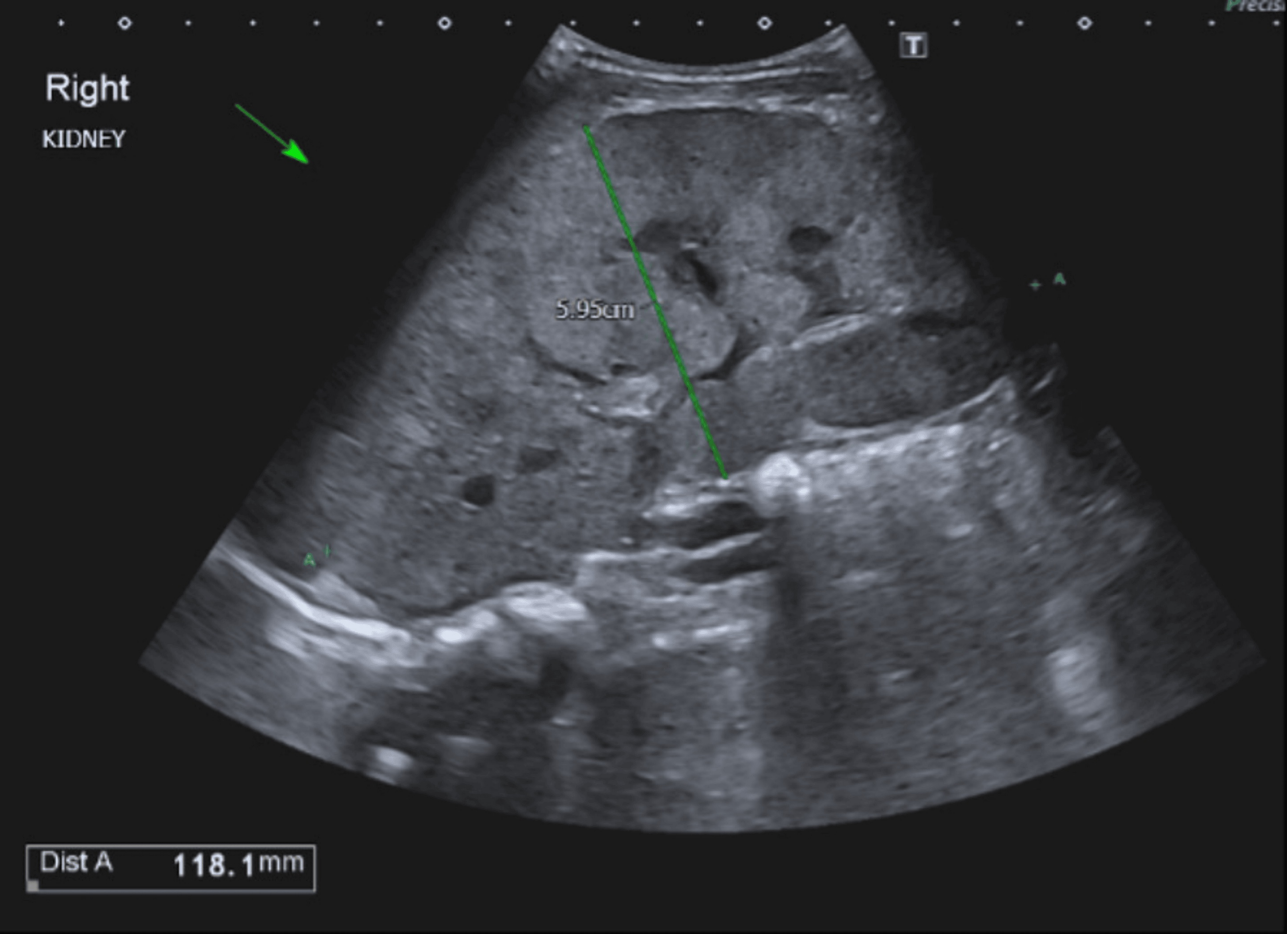
Abdominal ultrasonography of right kidney with largest width measurement of 5.95 cm and length (Dist A) of 11.81 cm

**Figure 2:**
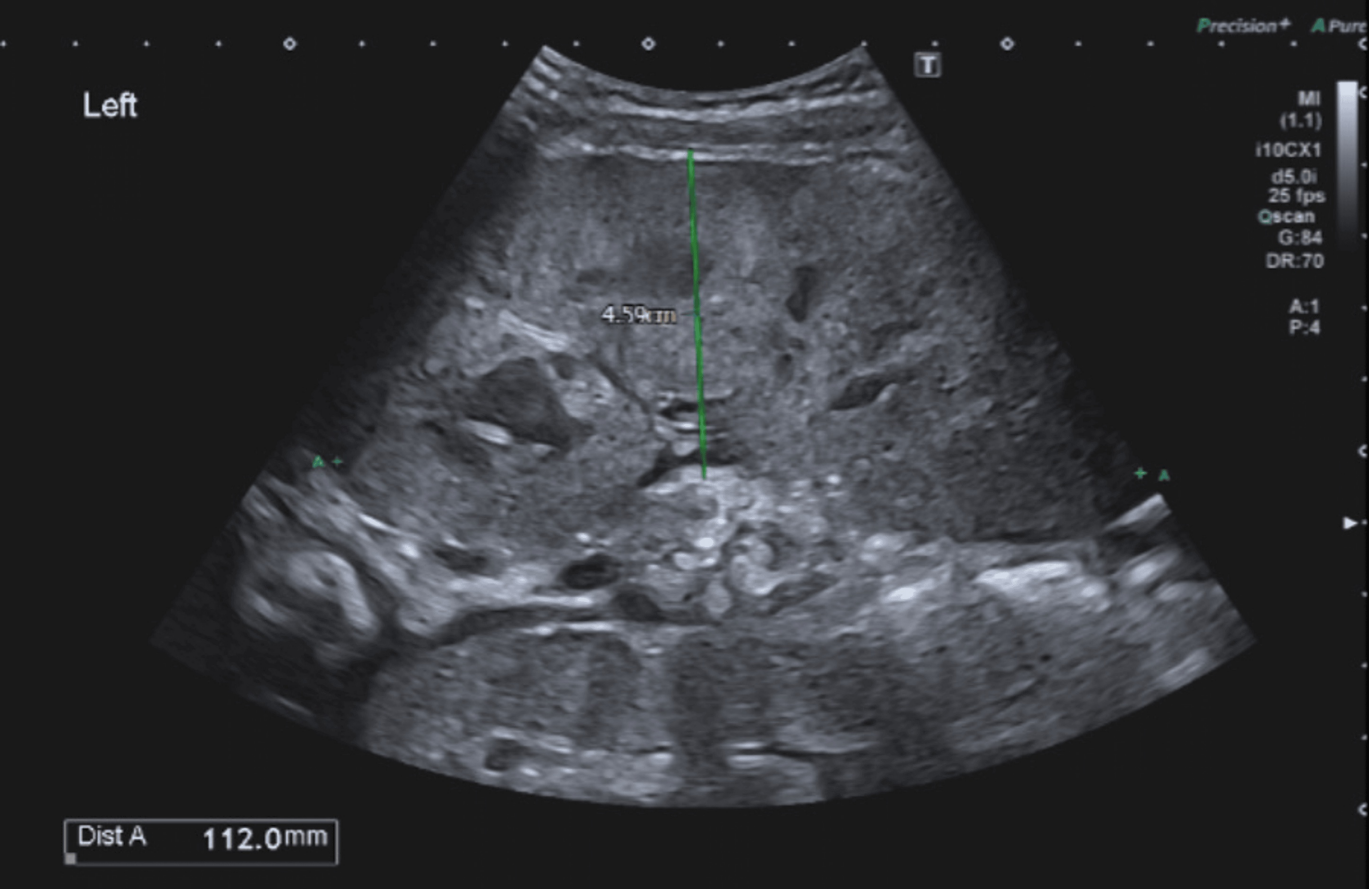
Abdominal ultrasonography of left kidney with largest width measurement of 4.59 cm and kidney length (Dist A) of 11.20cm.

**Figure 3:**
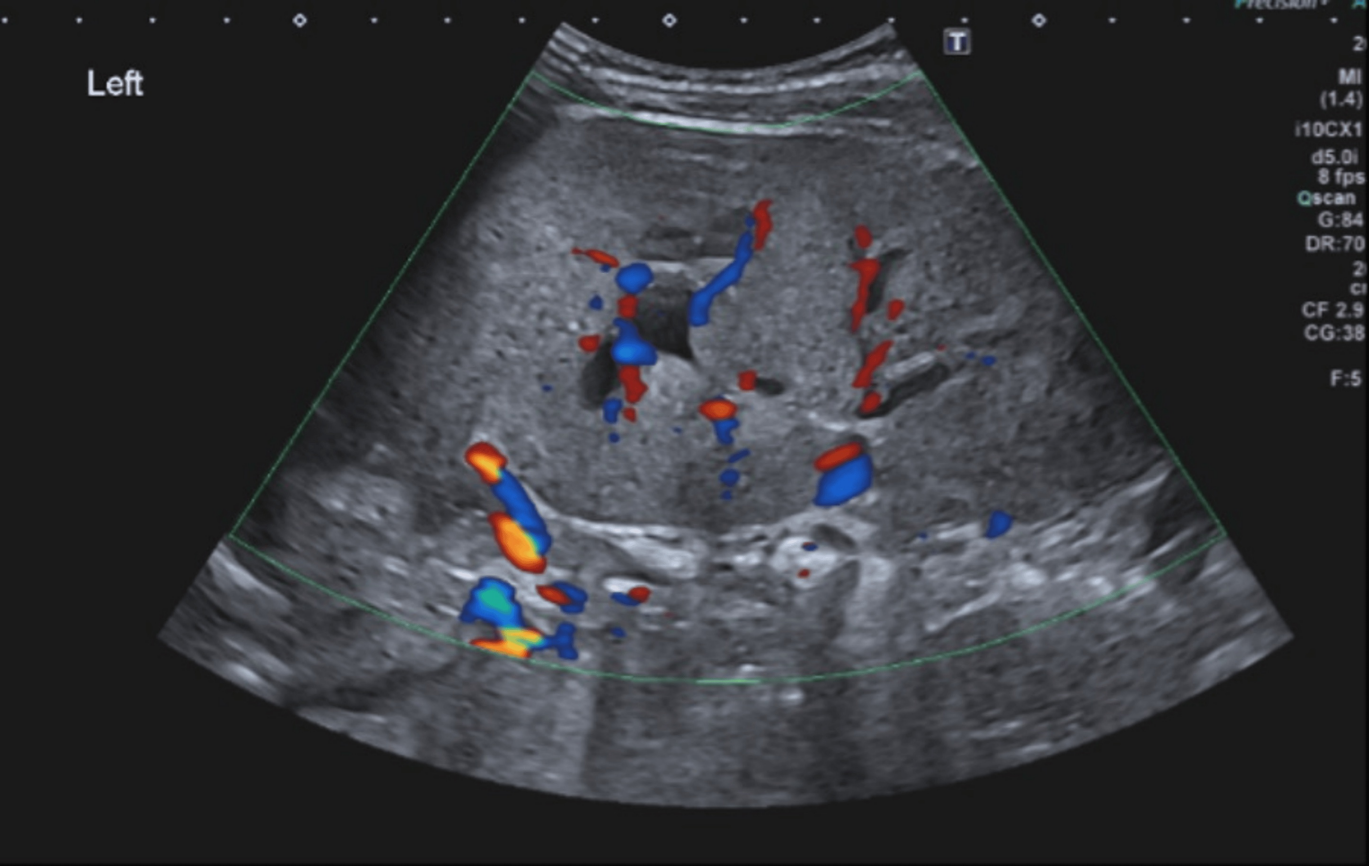
Abdominal Doppler ultrasonography of left kidney demonstrating no abnormality of the vascularity of the kidney

**Figure 4:**
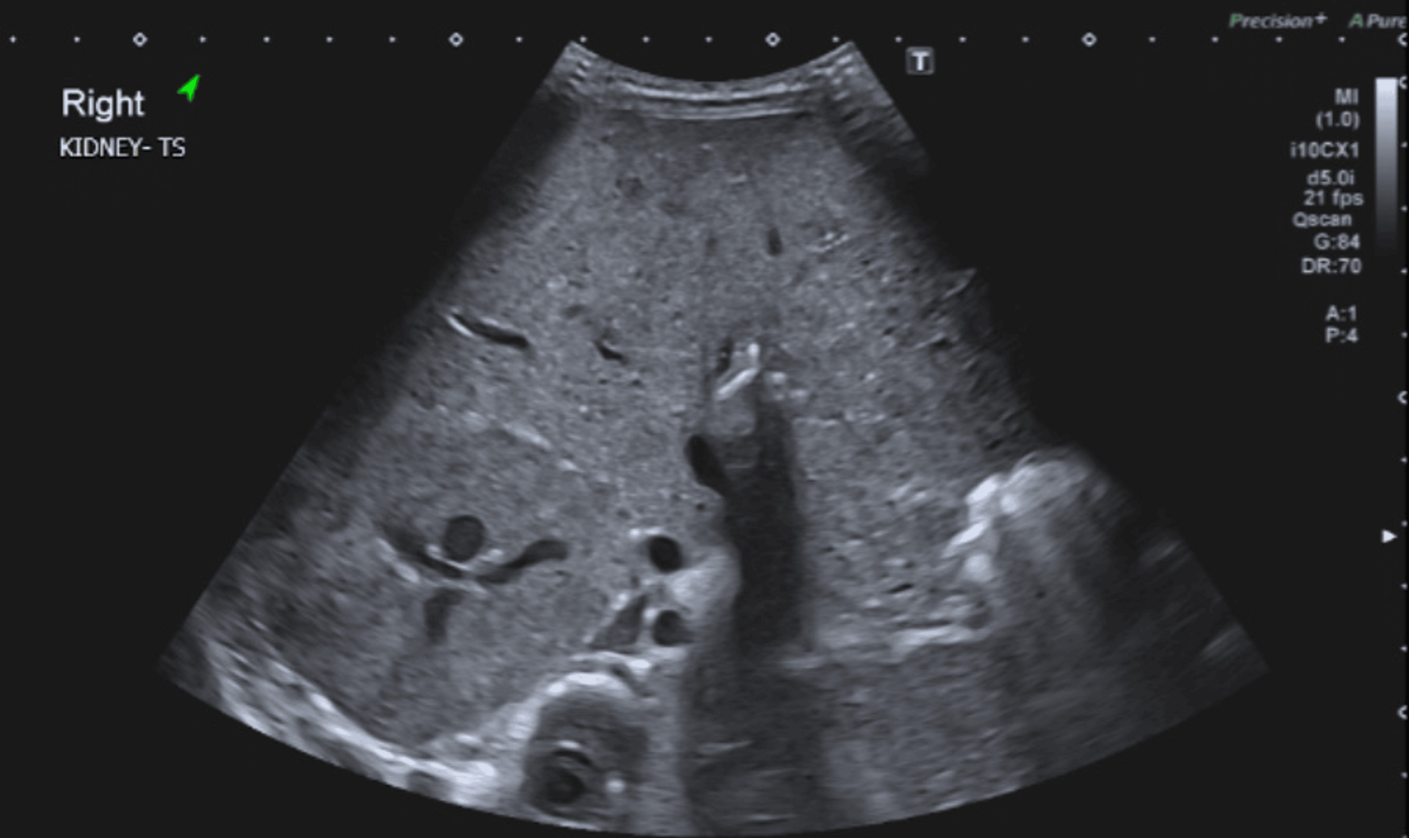
Abdominal ultrasonography, transverse cut through right kidney demonstrating loss of corticomedullary differentiation

Chest X-ray was normal without evidence of mediastinal involvement. Ultrasonography of the abdomen revealed bilateral massively enlarged kidneys measuring 11.81 cm in right kidney and 11.2cm in left kidney (normal sonographic kidney length in one-year-old female child: median 6.4 cm and normal range between 2.5th centile to 97.5th centile of 5.35cm - 7.55cm [5]), with parenchymal echogenicity and complete loss of corticomedullary differentiation as shown in Figures 1-4. Liver and spleen were not significantly enlarged. 

Flow cytometry was performed on a peripheral blood sample, and confirmed a precursor B-cell ALL (B-ALL) phenotype, positive for CD19 (low to intermediate), CD10 (low), CD45 (low), CD20 (dim), HLA-DR (up to intermediate), and CD13 (dim to low). 

The patient was initially commenced on intravenous (IV) fluids for hyperhydration at 3 L/m²/day and allopurinol 100 mg/m^2^ to prevent tumour lysis syndrome. Due to fevers, she was also started on broad-spectrum antibiotics as per the febrile neutropenia protocol. Her IV fluids were reduced to 2 L/m²/day, due to periorbital puffiness without signs of systemic fluid overload. She received a blood transfusion with furosemide cover, due to her haemoglobin level being lower than 70 g/L. The allopurinol was switched to rasburicase, which was subsequently stopped after five days. 

She underwent a bone marrow aspiration, which confirmed B-ALL, and cerebrospinal fluid analysis showed no involvement. A central line (Portacath) was inserted and commenced on multi-agent chemotherapy as per the standard of care in the United Kingdom for ALL with a four-drug induction (National Cancer Institute (NCI) high risk based on WBC) with dexamethasone, daunorubicin, vincristine, and PEG-asparaginase. 

Her renal function, fluid balance, blood pressure, and urine output remained normal throughout her admission. The size of the kidneys resolved clinically, and she did not require repeat imaging or biopsy. A repeat urine microscopy one month after presentation showed normalisation of WCC and RBC counts, measuring 0 and 2 × 10⁶/L, respectively.

Her recovery was complicated by norovirus-positive diarrhoea, requiring a prolonged hospital stay during the induction phase. She was subsequently discharged home on day 29 post presentation. At the end of induction (day 29), minimal residual disease assessment was undetectable by flow cytometry and molecular testing. She is now in the maintenance chemotherapy phase of treatment and continues in remission. 

## Discussion

B-ALL is a malignancy of lymphoblasts within the B-cell lineage. It is typically characterized by blast cells that are small in size with a high nuclear-to-cytoplasmic ratio [6]. The disease commonly involves the blood and bone marrow. 

This case demonstrated the common clinical features suggestive of an ALL diagnosis, including anaemia, thrombocytopenia, and organomegaly. The extramedullary finding of renal enlargement was further evaluated via ultrasonography, which suggested tumoural infiltration of the kidneys. Renal biopsy is generally not indicated in cases of leukemic kidney infiltration when the diagnosis has already been established through bone marrow aspiration or peripheral blood film. However, some studies have reported cases where nephromegaly with a normal blood film was further investigated with a renal biopsy, confirming leukaemic infiltration on histology [7]. 

Renal enlargement in childhood can result from various conditions, including polycystic kidney disease, renal vein thrombosis, kidney tumours, deposition diseases such as amyloidosis, and neoplastic infiltration [8]. Childhood ALL can present with signs and symptoms related to extramedullary involvement and should be considered in the differential diagnosis of a patient presenting with organomegaly or unexplained abdominal masses. 

Prada Rico et al. describe two potential causes of renal enlargement in paediatric ALL cases: (i) Diffuse or nodular infiltration: These cases tend to be asymptomatic and are usually discovered incidentally at diagnosis through ultrasound, and tend to involve the renal cortex and often are symmetrical and bilateral, (ii) Hypertrophy or hyperplasia of parenchymal cells: This represents a secondary response rather than direct leukaemic infiltration [3].

Previous studies have stated that leukaemic infiltration of the kidneys can be suspected on ultrasonography when findings include loss of corticomedullary differentiation, hyperechogenic enlarged kidneys, and other abnormalities such as hypoechogenic nodules or pelvicalyceal dilatation [3]. In our case, renal ultrasonography demonstrated loss of corticomedullary differentiation without any vascular abnormalities in either kidney, which is suggestive of leukaemic infiltration, consistent with findings reported in other case reports [9].

Mechanisms of acute kidney injury (AKI) in ALL include volume depletion (pre-renal), tumor lysis syndrome (TLS)-induced crystal nephropathies (due to the precipitation of uric acid, xanthine, and/or calcium phosphate crystals in the renal tubules), nephrotoxic drugs, renal vein or artery thrombosis and tumour-associated obstructive uropathy [10,11]. TLS can be a life-threatening complication of ALL and is relatively common in newly diagnosed children [12]. Early identification of patients at risk is therefore essential. Reported risk factors for TLS include a high WBC count, previous renal dysfunction, type of malignancy, age, and high tumour burden (stage/LDH) [12]. In our case, the markedly elevated LDH served as an indicator of extensive tumour burden, thereby placing the patient at increased risk of TLS and highlighting the importance of early recognition and preventive strategies.

Although nephromegaly was present in our patient, renal function was preserved and therefore did not contribute additional risk. However, the significant tumour burden, which placed the patient at high risk of TLS, justifies the initiation of prophylactic therapy, as timely intervention can significantly reduce morbidity and mortality [12,13]. The patient was commenced on allopurinol and subsequently switched to rasburicase, in line with current preventive strategies [13].

Leukaemic infiltration of the kidneys does not commonly lead to renal failure or hypertension. A retrospective study by Genc et al., involving 163 children with ALL, reported a 12% incidence of nephromegaly or increased renal echogenicity at initial presentation [14]. The study found that renal enlargement had no adverse effects on renal function, and there was no evidence of AKI before initiating chemotherapy. Multiple studies have shown that nephromegaly in ALL has no prognostic significance [9,15], with most reporting resolution of kidney enlargement following chemotherapy initiation. Although our case report also supports these observations, it also highlights the importance of TLS recognition and management to avoid renal injury in newly diagnosed patients with ALL. 

## Conclusions

This case of massive bilateral renal enlargement, accompanied by bicytopenia and leukocytosis on laboratory testing, highlights a rare presentation of ALL. Lymphoblastic infiltration of the kidneys is an uncommon extramedullary manifestation of leukaemia in paediatric patients, but should be considered in cases of unexplained nephromegaly or abdominal masses. Although limited evidence suggests that paediatric ALL patients with nephromegaly may not be at higher risk of renal dysfunction, careful monitoring of electrolytes, fluid balance and blood pressure remain of paramount importance. Early recognition and prevention of life-threatening complications such as TLS are crucial to optimising outcomes in children with ALL.
